# P-1821. Patient-Derived Airway Organoids for Modelling and Modulating *Rhinovirus* infection

**DOI:** 10.1093/ofid/ofae631.1984

**Published:** 2025-01-29

**Authors:** Alessandra Merenda, Nilofar Ehsani, Foteini Gkogkou, Johanna Pott, Taya Bezhaeva, Robert G J Vries, Sylvia F Boj

**Affiliations:** HUB Organoids B.V., Utrecht, Utrecht, Netherlands; HUB Organoids, Utrecht, Utrecht, Netherlands; HUB Organoids, Utrecht, Utrecht, Netherlands; HUB Organoids, Utrecht, Utrecht, Netherlands; HUB Organoids B.V., Utrecht, Utrecht, Netherlands; HUB Organoids B.V., Utrecht, Utrecht, Netherlands; HUB Organoids B.V., Utrecht, Utrecht, Netherlands

## Abstract

**Background:**

HUB’s patient-derived organoids (PDOs) are unique *in vitro* 3D models derived from adult stem cells that closely resemble the architecture and physiology of the tissue of origin. PDOs established from the airway epithelium are instrumental in investigating the pathogenesis of respiratory infections as well as testing strategies to counter such diseases. Because *in vivo* pathogens attack the luminal surface of the epithelium, HUB has established monolayers from airway PDOs, where the luminal side is accessible for mimicking host-pathogen interactions.

Viruses such as Rhinovirus type 16 (RV16) are among the most common respiratory pathogens affecting the airway tract. As an entry receptor, RV16 recognises the intercellular adhesion molecule-1 (ICAM-1) on the surface of epithelial cells, making it an attractive target for inhibiting RV16 infection.

**Methods:**

In this study we conducted experiments to assess the effect of an anti-ICAM-1 monoclonal antibody (mAb) on RV-16 infection. PDO-derived monolayers were pre-treated with different mAb concentrations before infection with RV16. Transepithelial electrical resistance (TEER) and monolayer integrity were monitored daily, and the viral titre was quantified at different time points post-infection (PI) by calculating the 50% tissue culture infectious dose (TCID_50_). Production of IP-10, an inflammatory cytokine released by the epithelium in response to infections, was also measured at 48 h PI.

**Results:**

Our data showed that viral titre increased over time when no pre-treatment was applied, indicating that RV16 could propagate in airway PDO monolayers. A significant decrease in viral titre was observed in monolayers treated with the highest ICAM-1 mAb doses. Such an inhibitory effect on viral titre was accompanied by a total suppression of IP-10 release upon mAb treatment, suggesting a potential role for ICAM-1 inhibition in immune response modulation during RV16 infections.

**Conclusion:**

Overall, this study not only revealed the value of PDO-derived monolayers as a platform for gaining insights into viral infection mechanisms but also demonstrated the potential of preclinically testing novel antiviral strategies. This findings have significant implications for the development of effective treatments for respiratory infections.

**Disclosures:**

**All Authors**: No reported disclosures

